# Influence of Selected Air Pollutants on Mortality and Pneumonia Burden in Three Polish Cities over the Years 2011–2018

**DOI:** 10.3390/jcm11113084

**Published:** 2022-05-30

**Authors:** Piotr Dąbrowiecki, Artur Badyda, Andrzej Chciałowski, Piotr Oskar Czechowski, August Wrotek

**Affiliations:** 1 Department of Allergology and Infectious Diseases, Military Institute of Medicine, 04-141 Warsaw, Poland; achcialowski@wim.mil.pl; 2 Polish Federation of Asthma, Allergy and COPD Patients Associations, 01-604 Warsaw, Poland; 3 Faculty of Building Services, Hydro- and Environmental Engineering, Warsaw University of Technology, 00-653 Warsaw, Poland; 4 Department of Quantitative Methods and Environmental Management, Faculty of Management and Quality Science, Gdynia Maritime University, 81-225 Gdynia, Poland; p.o.czechowski@wpit.umg.edu.pl; 5 Department of Pediatrics, The Centre of Postgraduate Medical Education, 01-813 Warsaw, Poland; awrotek@cmkp.edu.pl; 6 Department of Pediatrics, Bielanski Hospital, 01-809 Warsaw, Poland

**Keywords:** air pollution, ambient particulate matter, nitrogen dioxide, ozone, weather, modeling, mortality, morbidity

## Abstract

Poland has one of the worst air qualities in the European Union, particularly regarding concentrations of particulate matter (PM). This study aimed to evaluate the short-term effects of air pollution and weather conditions on all-cause mortality and pneumonia-related hospitalizations in three Polish agglomerations. We investigated data from 2011 to 2018 on a number of health outcomes, concentrations of PM_2.5_, PM_10_, nitrogen dioxide (NO_2_), ozone (O_3_), and selected meteorological parameters. To examine the impact of air pollutants and weather conditions on mortality and pneumonia burden, we identified optimal general regression models for each agglomeration. The final models explained <24% of the variability in all-cause mortality. In the models with interactions, O_3_ concentration in Warsaw, NO_2_, O_3_, and PM_2.5_ concentrations in Cracow and PM_10_ and O_3_ concentrations in the Tricity explained >10% of the variability in the number of deaths. Up to 46% of daily variability in the number of pneumonia-related hospitalizations was explained by the combination of both factors, i.e., air quality and meteorological parameters. The impact of NO_2_ levels on pneumonia burden was pronounced in all agglomerations. We showed that the air pollution profile and its interactions with weather conditions exert a short-term effect on all-cause mortality and pneumonia-related hospitalizations. Our findings may be relevant for prioritizing strategies to improve air quality.

## 1. Introduction

According to the “State of Global Air 2020” report prepared by the Health Effects Institute in 2019, air pollution was attributed to approximately 6.67 million deaths globally. It was identified as the fourth leading risk factor for early death, being surpassed only by high blood pressure, tobacco use, and poor diet [1]. Exposure to air pollution is associated with decreased lung function in children and adults [2,3,4,5,6].

An ongoing pandemic of Coronavirus Disease 2019 (COVID-2019) caused by the SARS-CoV-2 provided additional evidence for the impact of air pollution on human health. During lockdown, PM_2.5_ concentrations decreased on average by 29.7% in China and by 17.1% in Europe when compared with pre-pandemic daily concentrations in 2016–2019, which allowed for the avoidance of an estimated 24,200 premature deaths in China (between 1 February and 31 March 2020) and 2190 premature deaths in Europe (between 21 February and 17 May 2020) [7]. This analysis showed that substantial health benefits could be achieved once stringent emission control regulations are implemented [7].

The Great Smog of London of 1952 is one of the most recognized examples of the acute, short-term impact of air pollution on mortality. Stable foggy weather conditions gave rise to a massive increase in air pollution, resulting in approximately 4000 excess deaths [8]. Data on short-term health effects from exposure to air pollutants were presented in the 1990s, e.g., in the Air Pollution and Health, a European Approach (APHEA) project [9], and a decade later in the APHEA-2 project [10]. These studies found statistically significant adverse effects of sulfur dioxide (SO_2_), total suspended particles (TSP), nitrogen dioxide (NO_2_) and ozone (O_3_) on daily admissions for chronic obstructive pulmonary disease (COPD) and statistically significant adverse effects of NO_2_ on total, cardiovascular and respiratory mortality. In January 2017, a smog episode with significantly increased PM levels was observed in almost the entire territory of Poland (peak 1-h concentrations in Warsaw reached 243 and 337 μg/m^3^ for PM_2.5_ and PM_10_, respectively), leading to an increase in hospital admissions by 142%, 133%, and 33% for asthma, COPD, and atrial fibrillation, respectively [11]. These incidents prove the rapid, short-term effects of air pollution on health-related outcomes and highlight the interplay between air pollution profiles and specific meteorological conditions (i.e., temperature inversion, lack of wind or low wind velocity, lack of precipitation, and high atmospheric pressure), which trap the pollution in lower parts of the atmosphere and thus generate harmful health effects. 

Air quality in Poland is among the worst across Europe. Average annual levels of PM_2.5_ and PM_10_ in Poland are the highest and second highest, respectively, within the European Union [12], mainly due to the combustion of low-quality solid fuels, such as coal and wood, in domestic low-efficiency stoves/boilers or fireplaces [13]. In this paper, we focused our attention on coarse and fine particulate matter, i.e., PM_10_ and PM_2.5_ (particles with an aerodynamic diameter no greater than 10 μm and no greater than 2.5 μm, respectively), which in Poland is emitted primarily from residential, commercial and institutional sources (i.e., municipal and household sector). We also focus on NO_2_, which is a pollutant characteristic of transport (road transport in particular), posing a serious threat to air quality especially in large cities, and O_3_, which as a secondary pollutant is formed as a result of photochemical reactions occurring under the influence of, e.g., nitrogen dioxide in the ambient air [14,15]. According to data from 2019, concerning the emissions of air pollutants, inventoried and reported in accordance with the requirements of the United Nations Convention on the Long-Range Transboundary Transport of Air Pollutants [16] by the European Environment Agency [17], residential, commercial and institutional sources in Poland were responsible for over 43% and 40% of the total emission of PM_10_ and PM_2.5_, respectively. These sources also make a decisively dominant contribution to the emissions of polycyclic aromatic hydrocarbons (PAHs), which are easily adsorbed on the surface of particles [15,18]. In turn, road transport has a special presence in the emission of nitrogen oxides (NO_x_). In the case of other air pollutants, its role is not crucial, although the share of this sector in emissions of CO, PM_10_ and PM_2.5_, non-methane volatile organic compounds (NMVOC) or some metals is visible. It is worth bearing in mind, however, that the role of transport in shaping air quality is diversified, and its impact on the emission of pollutants, and thus their concentrations in the air, is particularly noticeable in large urban areas with high traffic intensity. Increased concentrations of pollutants typical for transport also occur outside cities, especially in the vicinities of busy roads with a significant share of heavy traffic (motorways, expressways, bypasses of the cities) [15]. The share of transport in the total NO_x_ emissions in Poland currently exceeds 41% [17]. The variability of air pollutant concentrations results not only from the activity of emission sources but also from various meteorological factors, such as wind strength, relative air humidity, type and intensity of precipitation, air temperature or intensity of solar radiation. These conditions have a significant impact on the dispersion, or dry and wet deposition, of both solid and gaseous pollutants, and in the case of solar radiation, they affect the intensity of photochemical reactions, as a result of which, for example, tropospheric ozone is formed. More on this subject can be found in the studies by Majewski et al. [19] or Ćwiek and Majewski [20]. 

The permissible concentrations of PM_10_, and to a lesser extent PM_2.5_ [21], regulated on the basis of the EU Directive on ambient air quality and cleaner air for Europe [22], are still quite commonly exceeded in Poland. In some large cities (including Warsaw and Cracow), NO_2_ levels are regularly exceeded. In some parts of Poland (especially in the west), the problem of not meeting the permissible levels of ozone also occurs [15].

Recently published results of the study by Kuzma et al. [23] proved the significant impact of air pollution on life expectancy. It was demonstrated that in Poland, even in the regions with relatively low air pollutants concentrations, the adverse effect of exposure to particulate matter can be seen. The relative risk (RR) of cardiovascular deaths for an increase in fine particulate matter (PM_2.5_) concentrations of 10 µg/m^3^ was at the level of 1.07 (95% confidence interval (CI): 1.02–1.12; *p* = 0.01); however, this effect was observed only in the male population. The study has also shown that an increase in SO_2_ concentration of 1 µg/m^3^ (RR = 1.07, 95% CI: 1.02–1.12; *p* = 0.005) and a decrease in temperature of 10 °C (RR = 1.03, 95% CI: 1.01–1.05; *p* = 0.005) were associated with an increase in the number of daily deaths, and in this case, no gender differences were noticed. In the research undertaken in 2017 in the Warsaw Metropolitan Area by Holnicki et al. [24], the impact of PM, NO_x_, SO_2_, CO, C_6_H_6_, BaP and heavy metals on the risk of mortality and the disability-adjusted life years index (DALY) was considered. In this study, it was predicted that the local emissions cause approximately 1600 possible deaths and 29,000 DALYs per year. About 80% of the health burden was due to exposure to fine particulate matter (PM_2.5_). Mobile and surface sources accounted for 46% and 52% of total DALYs, respectively. The study also provides information on the relative importance of the various air pollutants causing health hazards and information on the health effects they cause. For both attributable and DALY deaths, PM_2.5_ caused almost the entire health burden, and for DALY, most of the health burden from PM_2.5_ was related to non-accidental mortality [24,25]. Thus, it seems that the most important air pollutant in the case of non-accidental mortality is PM_2.5_. These results were comparable with the data on premature deaths reported by the European Environment Agency. Khomenko et al. [26] demonstrated the assessment of the PM_2.5_ and NO_2_ influence on natural mortality of adult residents (aged ≥ 20 years) in 969 cities and 47 greater cities in Europe. Researchers estimated the annual burden of premature mortality that could be prevented if the WHO Air Quality Guidelines (WHO AQG) (in force before September 2021, i.e., 10 and 40 μg/m^3^ for annual concentration of PM_2.5_ and NO_2_, respectively) were met and if air pollutant concentrations were lowered to the lowest values measured in 2015 in European cities (i.e., 3.7 μg/m^3^ for PM_2.5_ and 3.5 μg/m^3^ for NO_2_). As the author’s results show, compliance with the WHO AQG may prevent 51,213 (95% CI: 34,036–68,682) deaths per year in the case of PM_2.5_ exposure and 900 (95% CI: 0–2476) in case of exposure to NO_2_. Reducing the concentrations to the lowest measured level could prevent 124,729 (95% CI: 83,332–166,535) and 79,435 (0–215,165) deaths per year due to reduced exposure to PM_2.5_ and NO_2_, respectively. The highest mortality burden of PM_2.5_ was estimated for cities in the Po valley (northern Italy), Poland and Czechia, while the highest NO_2_ fatality burden was estimated for large cities and capitals in Western and Southern Europe. In the case of a new WHO AQG [27], similar calculations would be expected. According to the analyzes performed by Nazar and Niedoszytko [28], increased concentrations of air pollutants in Poland are associated primarily with an increased risk of the total number of deaths and deaths due to respiratory diseases but also with a higher incidence of respiratory diseases (including bronchial asthma, lung cancer, and in recent years also COVID-19). The authors also point to effects in the form of reduced forced expiratory volume within the first second of expiration (FEV_1_) and forced vital capacity (FVC). Similar results (however, limited to one agglomeration) were presented in the results of the study by Czechowski et al. [29]. It has been shown that air pollution and climatic conditions in the Tricity (an agglomeration located in northern Poland, on the Baltic coast) exacerbate the symptoms of bronchial asthma, also increasing the number of exacerbations of chronic obstructive pulmonary disease (COPD) and pneumonia.

In this study, we investigated the impact of day-to-day variations in air pollution levels and meteorological conditions on all-cause mortality and pneumonia-related hospitalizations as indicators of acute health effects of air quality. Using general regression models (GRM), we analyzed data collected between 2011 and 2018 from three major agglomerations in Poland and identified the negative health effects of air pollutants exposure. GRM uses the concept of general linear models (GLM), enabling it to capture the non-linearity of the impact of cause-and-effect relationships at the stage of non-linear link function as well as through the interactions of independent factors. The identification of interactions is a particularly important element of the presented research. GRM represents a group of models widely used for the evaluation of complex experimental systems consisting of qualitative and quantitative data expressed in various scales. An important advantage of a general linear model is its ability to describe non-linear relationships between variables due to the application of appropriate transformations of predictive factors and the use of substitution of z-score standardized variable methods. These types of models have previously been used to identify cause–effect relationships between air pollutant concentrations and selected phenomena (including health effects) [30,31,32,33,34]. In our study, the curvilinear relationships between variables were also described through the use of appropriate transformations of the predictors as well as the application of substitution methods using a standardized variable and a series of selected transformations of linearizing variables and through a non-linear linking function. Models were transformed into linear ones to show the most important relationships. One of the goals of this paper was also to define the periodic structure (such as seasons, months or years). Therefore, we used models that allowed the identification of factors in the frequency domain in separate locations for the analyzed period 2011–2018. In each initial model, the dependent variable was the selected disease entity.

## 2. Materials and Methods

### 2.1. Materials

We analyzed data regarding Warsaw, Cracow, and the Tricity metropolitan area (Gdansk, Gdynia, Sopot) over the period 2011–2018. The following data were gathered: number of all-cause deaths and pneumonia-related hospitalizations (provided by the Polish National Health Fund; pol. *Narodowy Fundusz Zdrowia*); meteorological conditions (air temperature and humidity, atmospheric pressure, precipitation, wind speed) and air pollutant concentrations (PM_2.5_, PM_10_, NO_2_, O_3_) (taken from the respective Regional Inspectorates of Environmental Protection). Air quality monitoring stations in Poland, operating within the State Environmental Monitoring, are located in accordance with the criteria of representativeness set out in national and European legal acts (e.g., directive 2008/50/EC of the European Parliament and of the Council of 21 May 2008 on ambient air quality and cleaner air for Europe).

The system is being successively developed; therefore, the number of air quality monitoring stations and types of pollutants measured changed slightly during the period under the presented analyses. Overall, depending on the pollution and year, data from the following number of measuring stations were acquired:

- Warsaw: NO_2_—3 stations, O_3_—3–4 stations, PM_10_—3–4 stations, PM_2.5_—2–4 stations
- Cracow: NO_2_—3–4 stations, O_3_—1 station, PM_10_—4 stations, PM_2.5_—3–4 stations
- Tricity: NO_2_—9 stations, O_3_—3–4 stations, PM_10_—8–9 stations, PM_2.5_—1 station

Of all air quality monitoring stations operating in the cities, one station served as a traffic station, while the remaining ones were urban background stations. Ozone concentrations were measured only at background stations. For the purposes of the analyses, data from all measuring stations that operated in a given city were used, regardless of their functions. Such approach was adopted because of a relatively small number of stations in each city and, therefore, the inability to accurately assign specific fractions of the city’s population to each of the measuring stations—especially considering not all stations measure all types of pollutants, and in some cases, only one station in the whole city measures the concentration of particular pollutants (e.g., O_3_ in Cracow or PM_2.5_ in the Tricity).

**Figure 1:**
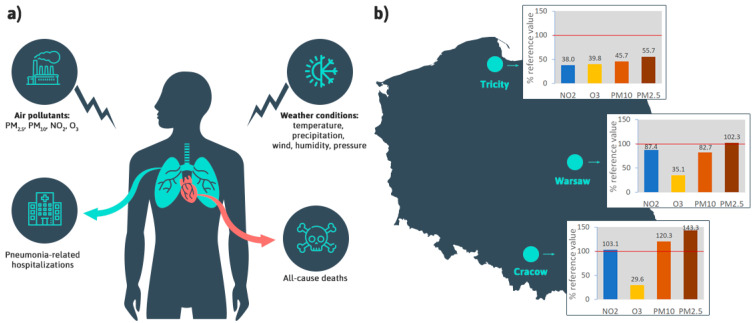
(**a**) The cause and effect relationship between the exposure to air pollution and associated weather conditions and health outcomes, including pneumonia-related hospitalizations and all-cause deaths; (**b**) geographical location of agglomerations: Tricity, Warsaw, and Cracow with mean concentrations of air pollutants in 2011–2018, expressed as a percentage of the reference values (calendar year limit values of 40 μg/m^3^ for PM_10_ and NO_2_, 25 μg/m^3^ for PM_2.5_, and maximum daily eight-hour mean target value of 120 μg/m^3^ for O_3_ were applied according to air quality standards stated in the EU Ambient Air Quality Directive), red lines correspond to 100% [13].

To build models, daily data from particular areas were used. If necessary, daily means or sums were calculated, depending on the data characteristics to reflect a day-to-day variability. Data flow and geographical locations of investigated agglomerations in Poland are shown in Figure 1.

### 2.2. Methods

#### 2.2.1. Data Collection, Aggregation and Analyses

All input data (air pollutants concentrations and meteorological parameters) were collected as 1-h mean values. For the purpose of the analyses, i.e., identification of factors and interactions, they were transformed to 24-h averages or, as in the case of atmospheric precipitation, to a 24-h sum. Data were subjected to a detailed quality analysis involving, if possible, a stage of interpolation of missing data as well as a detailed, multi-stage analysis of the causes of irregularities based on robust estimators (DFITS, Cook’s distance, Mahalanobis distance and classic Grubb’s and Rosner’s tests). Missing data were either interpolated with appropriate methods or removed by cases or variables.

Aggregation to daily data and data collection over a period of nine years determine the time horizon of conclusions. The models are long-term (nine years). Aggregation to daily data allows conclusions to be drawn in a shorter period of time, e.g., weekly, monthly or split up into seasons. This allows for the identification of regularities in the daily cycle (24-h), which is key for photochemical changes in air pollutant concentrations. Both the data and, therefore, the models used in the study do not enable estimation within a very short period of time, i.e., the daily cycle. Conclusions resulting from this study should be considered long-term, allowing the identification of regularities in shorter periods of time outside the daily cycle.

#### 2.2.2. General Linear Models (GLMs)

To determine and characterize the statistically significant effects of single variables along with their interactions on the investigated health outcomes, we identified statistical models belonging to the family of GRM (general regression models).

GRM uses the concept of the general linear models (GLM) family (e.g., GAM, GDA, PLS), enabling the capture of the non-linearity of the impact of cause and effect relationships at the stage of non-linear link functions as well as through the interactions of independent factors. The identification of interactions is a particularly important element of the presented research. These models, through a non-linear linking function (Gauss, Gamma, log, Poisson, etc.), allow modeling non-linear relationships between dependent variables and predictors on the basis of linearization of the variables’ distribution. This is a slightly different approach to the non-linear identification of cause-and-effect relationships such as the Box–Jenkins methodology, classic econometric, and ARIMA family models, etc.

As a result, complex equations describing non-linear functions are transformed into simpler ones of linear character. To illustrate, in the general linear model, a response variable *Y* is linearly associated with values on the *X* variables, while the relationship in the generalized linear model (GLM, GRM) is assumed to be:

$$Y=g\left(b_{0}+b_{1}\cdot X_{1}+...+b_{m}\cdot X_{m}\right)$$

where *g*(...) is a function. Formally, the inverse function of *g*(...), named, e.g., *g_i_*(...), is called the link function, so that:

$$g_{i}\left(muY\right)=b_{0}+b_{1}\cdot X_{1}+...+b_{m}\cdot X_{m}\;$$

where *muY* stands for the expected value of *Y*.

In the case of generalized additive models (GAMs), we can combine the notation of additive models with generalized linear models to derive the notation of GAMs as:

$$g_{i}\left(muY\right)=S_{i}\left(f_{i}\left(X_{i}\right)\right)$$

It means that the purpose of generalized additive models is to maximize the quality of prediction of a dependent variable *Y* from various distributions by estimating unspecific (non-parametric) functions of the predictor variables that are “connected” to the dependent variable via a link function.

Identification of the negative health effects of air pollutant exposure was based on the constructed GRM. It enables studying complex experimental systems and can include both qualitative and quantitative variables. This is particularly important when analyzing data expressed in different measurement scales. An important advantage is also the ability to describe curvilinear relationships between variables.

#### 2.2.3. Identification of the Periodic Structure

One of the goals of this work was to identify the periodic structure (e.g., seasons, months, years). Thus, we used the models that allowed us to identify the factors in the frequency domain in separate locations for the analyzed period of 2011–2018. In each initial model, a dependent variable was a selected disease entity. In addition, to take into account the abovementioned non-linearities with the strongest non-linear relations, the models include variables identifying the strongest periods of these relationships.

#### 2.2.4. Model’s Identification and Diagnostic

In order to identify the optimal final version of GRM, a stepwise forward regression method was used among others. It enables a selection of factors that have a statistically significant (*p* ≤ 0.05) impact on the dependent variable.

In contrast to corresponding methods, GRM measures the effects of interactions between two or more variables [35]. In our study, to ensure model simplicity, we limited interactions to two variables only. In the first step of a multistep process of model building, GRM was identified and the effects of single variables on a dependent variable were determined. Step 2 included the identification of models with interactions, whereas step 3 included the estimation of the significance of single variables (based on significance estimation of interactions using F-distribution).

The results of the final model identification include, among others, the presentation of the number of occurrences in individual interactions between air pollutants concentrations and meteorological parameters. Indeed, the number of occurrences is not the most accurate indicator of the importance of an effect of the given pollutant concentration on the disease. It is one of the elements of the weighting system that enables estimating the proportions of the strength of the effect of concentration based on the F-statistic, which precisely measures the strength of the effect and, thus, the importance of the factor. The number of variable occurrences was used to build a weighting system to estimate the strength of the effect of each variable separately. This strength is determined as the product of the number of occurrences (weighting system between variables) and the value of the F-statistic (proportions within interactions), assuming equal contribution of two factors in the interaction. Similar approaches and methods of data analysis were presented by Ratajczak et al. [36] and Wrotek et al. [37].

Pareto charts, being the standard method for showing the results of the model identification process in descending order based on the F, t, or t^2^ statistics, were drawn at each step of model identification. They show the strength of effects of single variables (Pareto A), interactions (Pareto C), and final estimation of single variables based on the model with interactions (Pareto B). Only statistically significant (*p* ≤ 0.05) variables were presented.

In each initial model, health outcome represented the dependent variable. Independent variables were as follows: year, season (summer season, from April to September; winter season, from October to March), quarter, month, and quantitative variables describing meteorological conditions and concentrations of air pollutants.

To identify the optimal (final) GRM, a forward stepwise approach was applied. Models were evaluated using an adjusted coefficient of determination (adjusted *R*^2^), describing the part of the total variation, which is explained by all statistically significant exogenous factors related to the model. The Pareto chart shows, in turn, the effects (arranged from the strongest to the weakest factor) statistically significantly associated with the endogenous variable, among all the effects that explain the total variation in the dependent variable (within *R*^2^). This relationship applies to both graphs with and without interactions.

All assumptions of applicability were tested (collinearity with the covariance matrix and in the case of interactions, homoscedasticity of variance with F and Bartlet tests, compliance of the random component with the normal distribution using the Shapiro–Wilk–Francis test with Royston’s correction). In addition, stationarity was tested with the Ljung Box Q test and the Durbin–Watson test, and the structure of partial correlations and autocorrelation was examined in detail. Only the models meeting the assumptions were considered final models.

Data pre-processing and calculations were performed in Statistica version 13 (TIBCO Software Inc., Palo Alto, CA, USA) and dedicated EDM Eco Data Miner version 1.09 (AirAnalyticsTech, Warsaw, Poland).

## 3. Results

### 3.1. Air Pollution Profile in Warsaw, Tricity, and Cracow in 2011–2018

**Figure 2:**
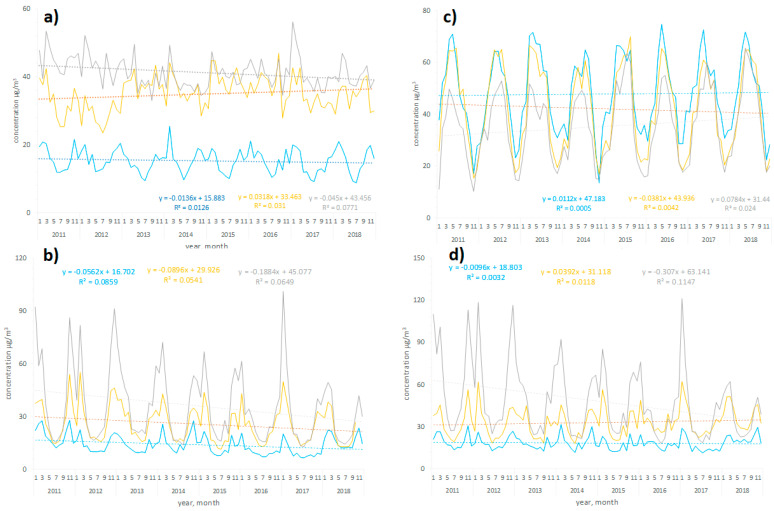
Daily concentration levels of air pollutants: (**a**) nitrogen dioxide (NO_2_); (**b**) ozone (O_3_); (**c**) particulate matter PM_2.5_; (**d**) particulate matter PM_10_ in analyzed agglomerations: Warsaw (in yellow); Cracow (in grey); Tricity (in blue), from 2011 to 2018. Linear regression curves are shown as dotted lines. Corresponding equations were also indicated.

Weather conditions, air pollutant profile and demographic characteristics were investigated in three agglomerations representing diverse geographical locations (Table 1 and Table 2). Cracow had the worst air quality, with the annual mean values of NO_2_, PM_10_, and PM_2.5_ exceeding the EU limits [22] (Figure 1, Table S1). This situation resulted from two groups of factors, the first of which was the intense emission of air pollutants from solid fuel (coal and wood) heating devices commonly used in the city. The second problem is the topography of Cracow—the city is located in the valley of the Vistula River, surrounded by hills. As a result, it is difficult to exchange air masses with the neighboring areas, and pollutants accumulate in the city. The problem of high air pollutant concentrations in Cracow, especially in the context of particulate matter and the PM-bound PAHs, has been partially resolved as a result of the implementation of the complete ban on the combustion of solid fuels in the municipal and household sector in the city from 2019. However, traffic-related air pollutants emissions and pollutants from towns surrounding Cracow, where similar restrictions have not been implemented, remain the source of poor air quality in the city. In Warsaw, only the mean annual PM_2.5_ concentration reached the EU threshold (Figure 1, Table S1). The lowest levels of NO_2_, PM_10_, and PM_2.5_ were observed in the Tricity; the mean annual concentrations were below the EU threshold (Figure 1, Table S1). Concentrations of air pollutants in 2011–2018 in the investigated agglomerations are shown in Figure 2. Seasonal changes in air pollution profiles were observed for PM_10_ and PM_2.5_, with peak concentrations in the winter season, and for O_3_, with peak concentrations in the summer season.

### 3.2. Models for All-Cause Deaths

The results of the final model identification revealed different profiles of variables (and their interactions) affecting mortality in the three investigated agglomerations (Table 3). In the table, the number of occurrences in individual interactions between selected air pollutants concentrations and meteorological parameters as well as their influence on the number of all-cause deaths were presented. Data on air pollutant levels and weather conditions were obtained for the models for each location separately. Numbers represent the number of times the variable was present in the final models. Statistically significant interactions and single variables included in the final models are shown and ordered by decreasing the strength of the effect.

The number of variable occurrences is used to build a weighting system to estimate the strength of the effect of each variable separately. This strength is determined as the product of the number of occurrences (weighting system between variables) and the value of the F-statistic (proportions within interactions), assuming equal contribution of two factors in the interaction. It is similar to the estimation of the proportions of success and failure in the model of the minimum sample size, where, without knowing the actual proportions, the worst possible option (the highest variance) of equal contribution of both quantities is assumed.

The models for Warsaw best explained the day-to-day variability in the number of deaths. The most complex description of outcomes was found for Warsaw, and relatively the simplest profile of mutual interactions was reported for the Tricity (25, 15, and 13 interactions of variables were statistically significant for Warsaw, Cracow, and the Tricity, respectively). The profile of air pollutant impact was distinct in the three agglomerations. The effect of pollutants was the most prominent in Warsaw (the variables occurred 8 times in the final model), followed by Cracow (6 times), and the Tricity (3 times). In Warsaw and Cracow, all investigated air pollutants were proven impactful, whereas in the Tricity, only PM_10_ and O_3_ were statistically significant.

**Figure 3:**
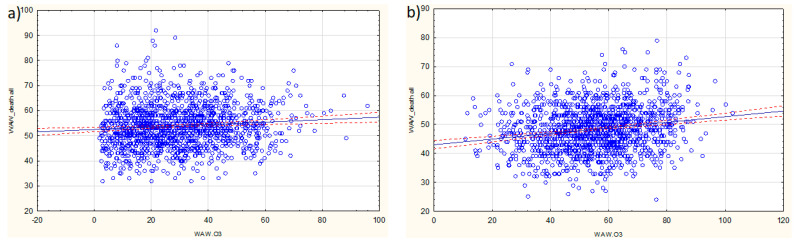
Association between daily ozone (WAW.O_3_) concentrations and mortality in Warsaw (WAW_death all): (**a**) winter season; (**b**) summer season. Regression line is shown in blue, and 95% confidence intervals are shown as dashed red lines.

For Warsaw, in a model without interactions, an increase in O_3_ concentration of 10 µg/m^3^ was associated with an increase in daily mortality by an average of 0.9% in the winter season (Figure 3a) and by 1.9% in the summer season (Figure 3b). Both figures show an actual relationship with the linear direction plotted for simplicity. Non-linearity for ozone, to which these figures refer, was implemented using a factor on a nominal scale (season), and for each season, the relationships were identified separately. This enables a clear interpretation of the non-linear relationship between exposure to ozone and the occurrence of the health effects.

The effect of ozone varies depending on the seasons (cold and hot) and the years of the study, as shown by the interactions in the models. It should be emphasized that the extent of O_3_ occurrence depends on concentrations of its precursors (e.g., NO_2_, CO, VOCs) and increases with solar radiation intensity and high temperatures [38]. Therefore, in our study, we analyzed the influence of O_3_ in the winter and summer seasons separately. In both seasons, an increase in O_3_ corresponded with an increased number of fatalities. 

For Cracow, NO_2_ concentration explained the 17.2% variability in all-cause mortality, followed by PM_2.5_ (7.0%) and O_3_ (6.7%) (Figure S1). An increase in NO_2_ concentration of 10 µg/m^3^ corresponded to an increase in mortality by 1.7%. For the Tricity, NO_2_ and PM_10_ concentrations accounted for the 4.9% and 3.4% variability, respectively, in all-cause mortality. 

**Figure 4:**
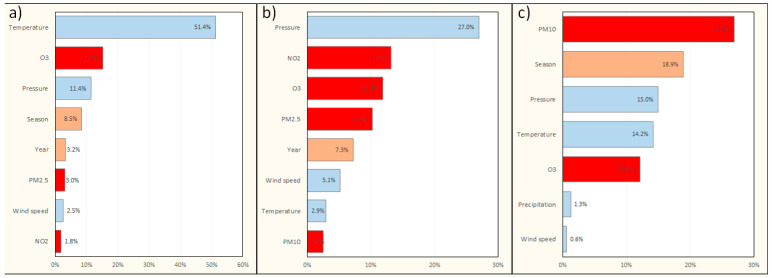
Pareto B graphs showing the impact of the most influential individual variables on the number of deaths in the final models with interactions. (**a**) model for Warsaw; (**b**) model for Cracow; (**c**) model for the Tricity. Factors associated with weather conditions are marked in blue, factors associated with air pollution are marked in red, and factors associated with seasonal/annual variability are marked in orange. PM_2.5_, particulate matter of size 2.5 μm or less; PM_10_, particulate matter of size 10 μm or less.

The impact of the most influential variables accounting for the number of deaths in the final models with interactions is shown in Figure 4. In Warsaw, O_3_ had the greatest impact among air pollutants on all-cause mortality. In Cracow, the impacts of NO_2_, O_3_, and PM_2.5_ were comparable. In the Tricity, PM_10_ was found to be the most influential factor.

In Cracow, the interaction between PM_2.5_ and wind speed had the greatest impact on mortality (Figure S2); a concurrent increase in PM_2.5_ concentration of 10 µg/m^3^ and wind by 1 m/s was associated with an increase in the number of all-cause deaths by 0.8%. In the Tricity, the interaction between O_3_ and PM_10_ was the most impactful. A simultaneous increase in O_3_ and PM_10_ concentrations of 10 µg/m^3^ corresponded to an increase in the number of all-cause deaths by 0.26%.

### 3.3. Models for Pneumonia-Related Hospitalizations

The results of the final model identification describing the effects of selected weather conditions and air pollutants on pneumonia-related hospitalizations are shown in Table 4. The models for Warsaw explained a higher fraction of day-to-day variability in the number of pneumonia-related hospitalizations than the models for Cracow and the Tricity. The most complex outcome models were obtained for Warsaw (26 statistically significant interactions of variables) and Cracow (27 variables), and a relatively simpler model was established for the Tricity (17 variables). Data on air pollutant levels and weather conditions were obtained for the models for each location separately. Numbers represent how many times the variable was present in the final models. Statistically significant interactions and single variables included in the final models are shown. 

The greatest impact of air pollutants was observed for Cracow (7 times), followed by Warsaw (6 times) and the Tricity (6 times). The most impactful air pollutants were NO_2_ for Warsaw and the Tricity and PM_10_ for Cracow.

**Figure 5:**
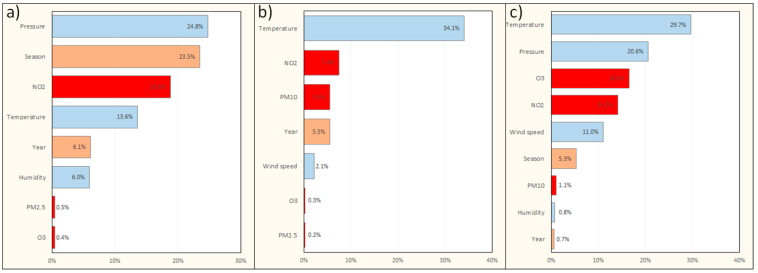
Pareto B graphs showing the impact of the most influential individual variables on the number of pneumonia-related hospitalizations in the final models with interactions. (**a**) Model for Warsaw; (**b**) model for Cracow; (**c**) model for the Tricity. Factors associated with weather conditions are marked in blue, factors associated with air pollution are marked in red, and factors associated with seasonal/annual variability are marked in orange. PM_2.5_, particulate matter of size 2.5 μm or less; PM_10_, particulate matter of size 10 μm or less.

In the models without interactions (Figure S3), the highest percentage of variability in the number of pneumonia-related hospitalizations among all pollutants was explained by NO_2_ concentration. An increase in hospitalizations per 10 µg/m^3^ NO_2_ increase was 4.5% for Warsaw, 7.7% for Cracow, and 11% for the Tricity. In the Tricity, PM_10_ and O_3_ also contributed to pneumonia-related hospitalizations. The impact of the most influential variables accounting for the number of pneumonia-related hospitalizations in the final models with interactions is shown in Figure 5.

The most influential pollutant-associated interactions that predicted variability in the number of pneumonia-related hospitalizations in Warsaw were NO_2_ with atmospheric pressure and NO_2_ with wind speed (Figure S4). An increase in NO_2_ concentration of 10 µg/m^3^ and concurrent increase in atmospheric pressure of 100 hPa or wind speed by 1 m/s were associated with an increase in pneumonia-related hospitalizations of 0.45% and 3.1%, respectively. In Cracow, NO_2_ and humidity interaction was the most influential among the pollutants. A rise in NO_2_ concentrations of 10 µg/m^3^ and in a humidity of 10% led to an increase in pneumonia-related hospitalizations of 1.3%. In the Tricity, an increase in NO_2_ and O_3_ concentrations (without seasonal breakdown) of 10 µg/m^3^ was associated with an increase in pneumonia-related hospitalizations of 0.58%. In an analysis involving division into seasons in the case of O_3_ concentrations, the impact of O_3_ was higher in the summer season and lower in the winter season.

### 3.4. Models for Pneumonia-Related Hospitalizations and for All-Cause Deaths with Interactions and Lags

Models with lag variables. The inclusion of delayed exogenous variables (concentrations of pollutants in each spatial location) was based on the total and partial autocorrelation function (ACF, PACF) and the Fourier spectral model. In the final models, the lag periods from 1 to 3 days back were considered due to the experience in building models for other regions of Poland so far. It does not seem appropriate to take into account longer delays in air pollution. Both our models and the conclusions contained in the literature are similar. Periods longer than 3 days do not appear to have an impact on the incidence of air pollutant concentrations.

The preliminary assessment of the degree of delay of each concentration was based on Ljung Box Pierce’s Q tests and the models used, inter alia, Durbin Watson (DW) statistics in the evaluation of stationarity. The normality of the random component distributions was tested with the Shapiro–Wilk–Francia test with the Royston correction, the Lilliefors test and the P-P analysis.

In further works, it is planned to use other models, including econometric and DLMN. GRM models allow for a broad and flexible assessment of the influence of selected factors on the incidence of death, regardless of the measurement scale of independent factors. These factors include pollutant concentrations, meteorological factors representing the fields, various types of periodic fluctuations related to the emissions of selected concentrations, and most importantly, taking into account the interactions between exogenous factors. GRM models have a non-linear linking function (Gamma and Gauss), which greatly increases their value in the study of non-linear phenomena. These are not classic linear models with linearized variables.

The obtained results (Table S2) after adding the delayed variables indicate a slight improvement in explaining the total variability (row “*R*^2^ (inter. And lags)”) from an additional 0.4% for the variable “deaths” in the Tri-City to 2.8% for the variable “pneumonia” in Warsaw. All final models are stationary models. The preliminary lag identification results from lag one to seven using GRM models are presented in Table S3.

## 4. Discussion

In this study, we showed that the interplay between ambient air pollution and weather conditions contributes substantially to the development of immediate negative health outcomes reflected by daily variations in mortality and pneumonia-related hospitalizations. Among the investigated pollutants, the impact of NO_2_ was especially pronounced, particularly for the pneumonia burden. 

PM_10_ and PM_2.5_ are recognized as key air pollutants significantly related to premature mortality [1,39]. The results of the European Study of Cohorts for Air Pollution Effects (ESCAPE) showed a higher overall mortality due to long-term PM_2.5_ exposure, with statistically significant associations also reported for individuals exposed to PM_2.5_ concentrations below the European threshold of 25 µg/m^3^ [40]. Short-term effects of PM_10_ on total mortality were reported in a multicity project; a 10 µg/m^3^ increase in daily PM_10_ corresponded to an increase in the number of deaths by 0.6% [41]. The observed city-specific heterogeneity in the exposure–response relationship was explained by factors characterizing the cities, i.e., climate, population, and geography [42]. In a meta-analysis of 33 studies from China addressing short-term effects of air pollution, a 10 µg/m^3^ increase in PM_2.5_ (PM_10_) was associated with a 0.38% (0.32%) increase in total mortality [42]. Data for China may be more relevant to our analysis, as PM concentrations in China are far higher than the Western European or North American averages and comparable to those in Poland [42]. In our study, the estimated short-term effects of daily PM_10_ concentrations on mortality were more profound than in the European or Chinese analyses; 2.1% and 2.6% increases in the number of deaths were reported per 10 µg/m^3^ PM_2.5_ increase for the Tricity and Warsaw, respectively. 

The profile of relationships between air pollutants and weather conditions in producing health outcomes varied between the three investigated agglomerations. This corroborates the findings of Katsouyanni et al. [41] and Samoli et al. [43], who found the city-specific heterogeneity in the relation between exposure to air pollution and mortality. These variations were attributed to differences in air pollution profiles, climate, and general health status of investigated populations [41,43]. In the Tricity, the relative impact of PM_10_ on mortality was equal to Warsaw and greater than in Cracow. This might suggest that even low PM concentrations can cause negative health outcomes, as reported previously [40,44]. Additionally, the high atmospheric pressure in the Tricity, which traps PM, might explain this observation. 

Within the time period of our study, in Warsaw and Cracow, average levels of PM_10_, PM_2.5_, and NO_2_ either exceeded or were close to reaching EU limits. Although PM concentrations are the most critical in Poland [12], in our study, NO_2_ was a principal contributor to pneumonia-related hospitalizations and exerted effects on all-cause mortality similar to those of PM and O_3_. As road traffic is a major source of NO_2_ emissions, the health impact of NO_2_ may be particularly visible in urban areas, where heavy traffic usually congeals in densely populated areas. Therefore, the estimated effect described in our study for the agglomerations might not reflect the country average. Additionally, we estimated the immediate day-to-day effect of air pollution on health outcomes. Longer-term effects of PM exposure could be greater than the immediate ones. According to Zanobetti et al. the acute effect of PM_10_ (per 10 µg/m^3^) for respiratory deaths was 0.74%; whereas, in the long-term, the effect increased to 4.2% and persisted for a month or longer [45]. Nevertheless, our study showed that the acute health outcomes associated with NO_2_ exposure might be worse than anticipated [40]. This finding might be relevant for shaping air quality management policies toward further restrictions for road traffic in densely populated areas [46].

In the ESCAPE project, no long-term effect of NO_2_ on natural-cause mortality was shown [40]. In contrast, in a meta-analysis of 19 studies, Faustini et al. demonstrated that an annual increase in NO_2_ of 10 µg/m^3^ was associated with a 4.1% increase in natural mortality [47]. The magnitude of NO_2_ effects on mortality was similar to that of PM_2.5_ [47]. In the short-term, a 0.3% increase in total daily mortality was reported per 10 µg/m^3^ NO_2_ [10]. It was highlighted that confounding effects of other pollutants are possible [10], as NO_2_ is proposed as a surrogate for PM [48]. In 17 Chinese cities (with average NO_2_ levels of 26–67 µg/m^3^), each short-term 10 μg/m^3^ increase in NO_2_ corresponded to a 1.63% increase in mortality [49]. The association remained significant when adjusted for PM [49]. Our findings, which showed an increase in daily mortality of 1.7% in Cracow (and of 3.9% in the Tricity) per 10 µg/m^3^ NO_2_ increase, are in line with the magnitude of short-term NO_2_ impact on mortality observed in China [49], where NO_2_ exposure was similar to that in our analysis. Our study also suggests that the effect of NO_2_ is independent of other air pollutants. Four major pollutants (PM_2.5_, PM_10_, NO_2_, O_3_) were used together to predict health outcomes, and the final models took into account all interactions between air pollutants and other possible confounders such as weather conditions or seasonal changes.

Our findings highlight the prominent impact of NO_2_ on pneumonia-related hospitalizations. Pneumonia is an important cause of deaths globally, especially in young children and the elderly. In 2016, lower respiratory tract infections contributed to 5.2% of total deaths [50]. In individuals with asthma, NO_2_ potentiates bronchial responsiveness [51] and triggers both allergen-dependent [52] and independent [53] eosinophilic inflammation. NO_2_ at ≥2 ppm concentrations were reported to disrupt the tracheobronchial epithelial monolayer [54] and to modify the severity of viral infections [55]. Investigations within the ESCAPE project on the long-term association of air pollution with respiratory infections in children showed that PM_10_ and NO_2_ significantly increased pneumonia incidence [56]. Most studies investigating the immediate effects of air pollutants on pneumonia burden pointed toward the role of PM [57,58,59,60]. In the short-term, exposure to PM between 2.5 and 10 μm in aerodynamic diameter (PMC) and PM_2.5_ was associated with an increased number of emergency hospitalizations for pneumonia of 3.3% and 1.7% per 10 µg/m^3^ increase in PMC and PM_2.5_, respectively [60]. In contrast, in our study, NO_2_ clearly had the greatest immediate effect on pneumonia-related hospitalizations in all investigated agglomerations and a NO_2_ increase of 10 μg/m^3^ was associated with up to an 11% increase in the number of daily pneumonia-related hospitalizations.

Environmental conditions are important modifiers for health outcomes associated with air pollutants. In particular, the effect of temperature is well-established [61,62,63]. Mutual interactions of ambient air pollutants and their interplay with weather conditions are the most evident for O_3_ as a secondary pollutant. The extent of O_3_ production is dependent on its precursor concentrations (e.g., NO_2_), and increases with solar radiation intensity and high temperatures [38]. Therefore, in this study, we analyzed the impact of O_3_ in the winter and summer seasons separately. In both seasons, an increase in O_3_ corresponded with an increased death toll. We identified the combined effect of the year and factors, which means that not only the passage of time does matter but also the passage of time and some factors during that time (in years) are significant. Season-dependent acute effects of O_3_ on mortality were shown previously by Gryparis et al. [64], who reported a positive association of O_3_ levels with mortality in the warm season and not in the cold season. 

Among other pollutant-weather interactions reported in our study, the effect of PM_2.5_ on mortality was directly related to wind speed. This finding is unexpected, as increasing ventilation has a diluting effect on PM [65] and stagnant, windless weather conditions were characteristic of severe smog episodes with high mortality rates [8,11,66]. 

This study has certain limitations. First, the data used for the models contained no individual characteristics (such as demographics or comorbidities), which could have an impact on the evaluated health outcomes. Such an approach, however, in which anonymized records from the National Health Fund (covering all public healthcare providers) were collected, enabled us to analyze large (and complete) sets of entries over eight years. Second, data on air pollution were taken from the only available central monitoring sites and, therefore, represent an average for a given agglomeration. It cannot be excluded that locally, the profile of air pollution, and thus individualized exposure, might be substantially different. Third, we investigated three agglomerations diverse in terms of weather conditions and air pollution profile. Corresponding data regarding less urbanized areas where traffic- and industry-related air pollution levels would be lower could provide additional sources of information on the relative role of particular pollutants.

## 5. Conclusions

To conclude, our study showed that an air pollution profile along with its interactions with weather conditions exert an immediate effect on all-cause mortality and pneumonia-related hospitalizations. A distinct pattern of interactions and magnitude of effects of particular pollutants were observed across three investigated agglomerations, demonstrating the importance of local microenvironments for generating health outcomes. The day-to-day variability in the number of deaths was best explained by the models created for Warsaw, where also the most complex description of outcomes was found (25 interactions). The relatively simplest profile of mutual interactions was reported for the Tricity (13 interactions). Similar findings were demonstrated in the case of pneumonia-related hospitalizations; however, the highest complexity of models was found in the case of both Warsaw (26 interactions) and Cracow (27 interaction), while in the Tricity, the model was relatively simpler (17 variables). 

Our analysis highlighted the key role of NO_2_, particularly in pneumonia-related hospitalizations. Increased concentrations of each of the considered air pollutants, however, were associated with a growing number of hospitalizations due to pneumonia and an increased number of premature deaths. In the case of rising numbers of premature deaths associated with each 10 µg/m^3^ increase in concentration, we found the highest influence of ozone in Warsaw (1.9% increase in daily mortality in summer season), PM_2.5_ in Cracow (0.8% increase) and NO_2_ and PM_10_ in Tricity (4.9% and 3.4% respectively). For pneumonia-related hospitalizations, the highest incidence growths were associated with each 10 µg/m^3^ NO_2_ increase in Warsaw (4.5%), Cracow (7.7%) and Tricity (11%). 

Our findings may be relevant for building strategies to improve air quality in countries where prioritization might be crucial to achieve prompt health effects. Our models could predict up to half of the day-to-day variability in health outcomes. Such an approach can be implemented locally as a part of a community alert system for planning outdoor activities.

## Figures and Tables

**Table 1:** Characteristics of the analyzed agglomerations in Poland.

Characteristic	Warsaw	Cracow	Tricity
Population ^†^	1,790,658	779,115	752,974
Population density, per km^2 †^	3462	2384	1811
Geographical location	Central lowlands	Foothills	Coastal plain
Weather conditions ^§^			
Temperature, °C	9.5 (9.0–10.1)	9.5 (9.0–10.1)	8.7 (8.1–9.3)
Air humidity, %	75.8 (73.8–77.9)	77.0 (75.8–78.3)	78.7 (78.2–79.3)
Wind speed, m/s	2.9 (2.8–3.0)	2.4 (2.4–2.5)	3.9 (3.7–4.0)
Precipitation, mm ^#^	616.9 (515.9–717.8)	699.8 (585.6–814.1)	619.1 (491.2–746.9)
Atmospheric pressure, hPa ^‡^	1003.5 (1003.2–1003.8)	988.1 (987.8–988.4)	1007.1 (1006.8–1007.4)
Air pollution ^§^			
PM_2.5_, µg/m^3^	25.6 (25.0–26.2)	35.8 (34.7–36.9)	13.9 (13.6–14.3)
PM_10_, µg/m^3^	33.1 (32.4–33.7)	48.1 (46.7–49.5)	18.3 (17.9–18.7)
NO_2_, µg/m^3^	35.0 (34.6–35.4)	41.3 (40.8–41.7)	15.2 (14.9–15.5)
O_3_, µg/m^3^	42.1 (41.4–42.9)	35.5 (34.8–36.2)	47.8 (47.1–48.4)
Number of evaluated health outcomes in 2011–2018			
All-cause deaths	149,418	59,127	62,771
All-cause deaths (per 100,000 inhabitants)	8404	7668	8,381
Pneumonia-related hospitalizations	45,046	20,165	12,350
Pneumonia-related hospitalizations (per 100,000 inhabitants)	2531	2612	1649

Gdańsk, Sopot, Gdynia; ^†^ data derived from Local Data Bank, Statistics Poland (valid on 31 December 2019); ^§^ based on data collected within this study (2010–2018), daily means (95% confidence intervals) are given; ^#^ annual sums are shown; ^‡^ actual local (unadjusted) atmospheric pressure values are shown. PM_2.5_, particulate matter of size 2.5 μm or less; PM_10_, particulate matter of size 10 μm or less.

**Table 2:** EU limit values and WHO Air Quality Guidelines (AQG) for considered air pollutants.

Air Pollutant	EU Limit Values *		Annual WHO AQG ^#^	
	μg/m^3^			
	Annual	Daily	Annual	Daily
PM_2.5_, µg/m^3^	25	n.a.	5	15
PM_10_, µg/m^3^	40	50	15	45
NO_2_, µg/m^3^	40	n.a.	10	25
O_3_, µg/m^3^	n.a.	120 ^†^	60 ^§^	100 ^‡^

* Limit values according to EU Directive on ambient air quality and cleaner air for Europe [22]; ^#^ WHO Air Quality Guidelines in force since September 2021 [27]; ^†^ Target value, maximum daily 8-h mean; ^§^ average of daily maximum 8-h mean O_3_ concentration in the 6 consecutive months with the highest six-month running-average O_3_ concentration; ^‡^ 8-h mean; n.a.—not applicable.

**Table 3:** Characteristics of the final models (with and without interactions) describing effects of air pollutants and weather conditions on the number of all-cause deaths.

	Warsaw	Cracow	Tricity
*R*^2^ (inter.)	24.0%	9.9%	12.7%
*R*^2^ (univ.)	19.7%	9.4%	11.2%
df	2800	2860	2888
Year	3	2	3
Season	4	1	1
NO_2_	2	2	0
O_3_	2	2	1
PM_10_	2	1	2
PM_2.5_	2	1	0
Pressure	2	2	2
Wind	2	1	1
Temperature	4	1	2
Humidity	1	2	0
Precipitation	1	0	1
Interactions:	O_3_ × temperature	Season × humidity	O_3_ × PM_10_
	Pressure × temperature	Pressure	Season
	PM_2.5_ × wind	PM_2.5_ × wind	Pressure × temperature
	Season × O_3_	NO_2_ × O_3_	Year × pressure
	Year × PM_10_	Pressure × temperature	Year × temperature
	NO_2_ × humidity	Year × PM_10_	PM_10_ × precipitation
	PM_10_ × precipitation	NO_2_ × humidity	Year × wind
	PM_10_ × temperature	Year × O_3_	
	Season		
	Season × NO_2_		
	Season × pressure		
	Year × temperature		
	Year × wind		
Single factors:	Season	Year	Season
	O_3_	Season	O_3_
	PM_10_	NO_2_	Year
	Year	Pressure	Temperature
	Temperature	Wind	Humidity
	Pressure	Temperature	NO_2_
		PM_2.5_	PM_10_
		O_3_	

*R*^2^ (inter.), adjusted coefficient of determination for models with interactions; *R*^2^ (univ.), adjusted coefficient of determination for models without interactions; df, degrees of freedom of the final model; PM_10_, particulate matter of size 10 μm or less; PM_2.5_, particulate matter of size 2.5 μm or less; pressure, atmospheric pressure; wind, wind speed; temperature, air temperature; humidity, air humidity.

**Table 4:** Characteristics of the final models (with and without interactions) describing effects of air pollutants and weather conditions on the number of pneumonia-related hospitalizations.

	Warsaw	Cracow	Tricity
*R*^2^ (inter.)	45.8%	29.8%	17.4%
*R*^2^ (univ.)	40.8%	26.1%	16.7%
df	2800	2860	2888
Year	5	5	1
Season	3	4	1
NO_2_	3	2	3
O_3_	1	1	2
PM_10_	1	3	1
PM_2.5_	1	1	0
Pressure	4	0	2
Wind	2	3	3
Temperature	4	5	3
Humidity	1	3	1
Precipitation	0	0	0
Interactions:	Season × humidity	Temperature × humidity	Pressure × temperature
	NO_2_ × pressure	NO_2_ × humidity	Season × O_3_
	NO_2_ × wind	Season	NO_2_ × O_3_
	Season × PM_10_	PM_10_ × temperature	Wind × temperature
	NO_2_ × temperature	Season × PM_10_	Pressure
	Pressure	Season × humidity	PM_10_ × wind
	Pressure × temperature	Season × temperature	NO_2_ × wind
	PM_2.5_ × temperature	Wind × temperature	NO_2_ × humidity
	Year × O_3_	Year × O_3_	Year × temperature
	Season × temperature	Year × temperature	
	Year × temperature	Year × PM_10_	
	Year × wind	NO_2_ × wind	
	Year	Year × PM_2.5_	
	Year × pressure	Year × wind	
Single factors:	Temperature	NO_2_	NO_2_
	NO_2_	Temperature	Temperature
	Season	Season	Season
	Wind	Wind	PM_10_
	Pressure	Pressure	O_3_
	Year	Year	Wind
			Pressure
			Year

*R*^2^ (inter.), adjusted coefficient of determination for models with interactions; *R*^2^ (univ.), adjusted coefficient of determination for models without interactions; df, degrees of freedom of the final model; PM_10_, particulate matter of size 10 μm or less; PM_2.5_, particulate matter of size 2.5 μm or less; pressure, atmospheric pressure; wind, wind speed; temperature, air temperature; humidity, air humidity.

## Data Availability

The data presented in this study are available upon request from the corresponding authors.

## Supplementary Materials

The following are available online at https://www.mdpi.com/article/10.3390/jcm11113084/s1, Figure S1: Pareto A graphs showing the impact of the most influential individual variables on the daily number of deaths for Warsaw (a), Cracow (b), and Tricity (c). PM_2.5_, particulate matter of size 2.5 μm or less; PM_10_, particulate matter of size 10 μm or less, Figure S2: Pareto C graphs showing the impact of the most influential (individual or their interactions) variables on the daily number of deaths for Warsaw (a), Cracow (b), and Tricity (c) in final models with interactions. PM_2.5_, particulate matter of size 2.5 μm or less; PM_10_, particulate matter of size 10 μm or less, Figure S3: Pareto A graphs showing the impact of the most influential individual variables on the daily number of pneumonia-related hospitalizations for Warsaw (a), Cracow (b), and Tricity (c). PM_2.5_, particulate matter of size 2.5 μm or less; PM_10_, particulate matter of size 10 μm or less, Figure S4: Pareto C graphs showing the impact of the most influential (individual or their interactions) variables on the daily number of pneumonia-related hospitalizations for Warsaw (a), Cracow (b), and Tricity (c) in final models with interactions. PM_2.5_, particulate matter of size 2.5 μm or less; PM_10_, particulate matter of size 10 μm or less, Table S1: Annual mean concentrations of air pollutants in Warsaw, Cracow and the Tricity, with annual limit values according to the EU guidelines. Table S2: Characteristics of the final models (with interactions and lags) describing effects of air pollutants and weather conditions on the number of all-cause deaths and pneumonia-related hospitalizations, Table S3: Preliminary lag identification results from lag 1 to 7 using GRM models. File S1. Brief description of the GLM (generalized linear model) and GRM (generalized regression model) link function models.
