# Supplementary material for: Influence of Selected Air Pollutants on Mortality and Pneumonia Burden in Three Polish Cities over the Years 2011–2018
Source: J Clin Med. 2022 May 30;11(11):3084. doi: 10.3390/jcm11113084 (PMC9181391; doi:10.3390/jcm11113084)
Supplement: Supplementary file 1 [file jcm-11-03084-s001.zip › Supplementary materials (Figures S1--S4).pdf]

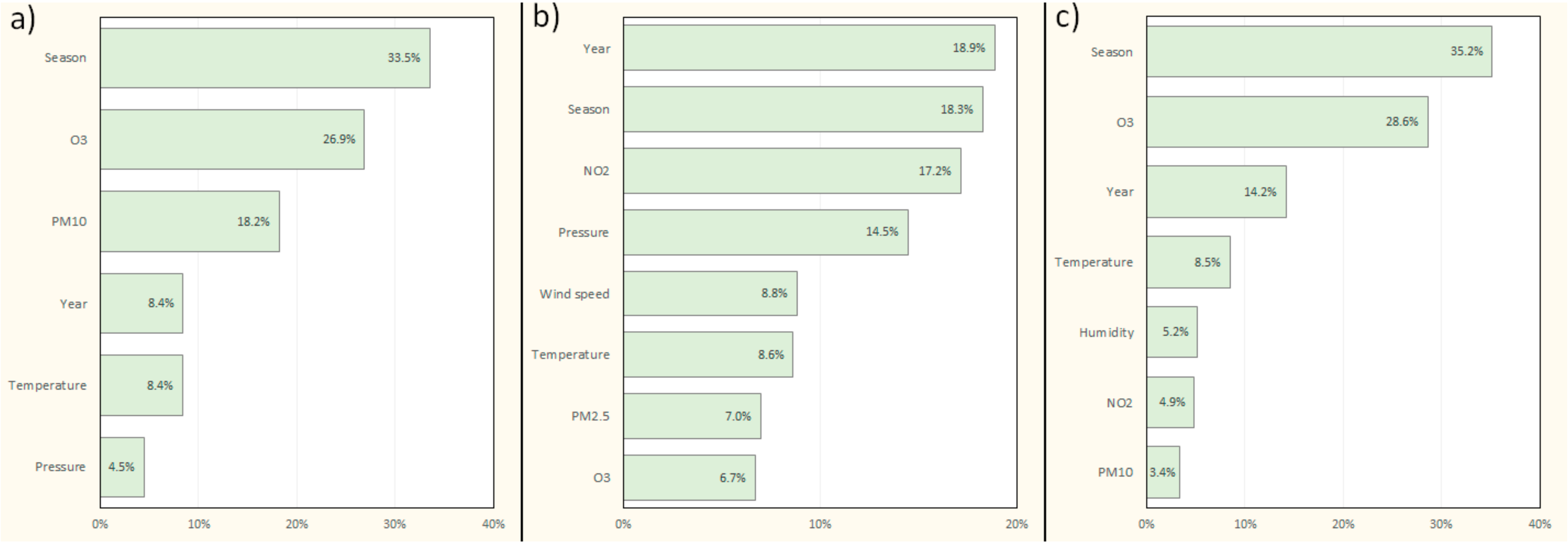

Figure S1. Pareto A graphs showing impact of the most influential individual variables on daily number of deaths for Warsaw (a), Cracow (b), and Tricity (c). $PM_{2.5}$, particulate matter of size 2.5 μm or less; PM_{10}, particulate matter of size 10 μm or less.

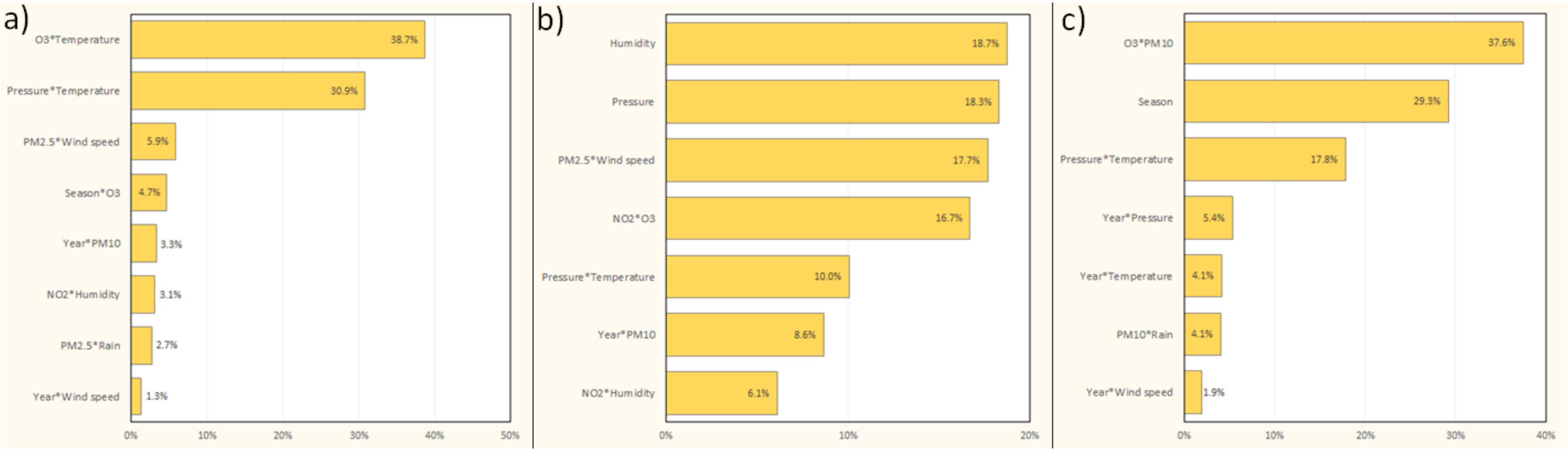

Figure S2. Pareto C graphs showing impact of the most influential (individual or their interactions) variables on daily number of deaths for Warsaw (a), Cracow (b), and Tricity (c) in final models with interactions. $PM_{2.5}$, particulate matter of size 2.5 µm or less; PM_{10}, particulate matter of size 10 µm or less.

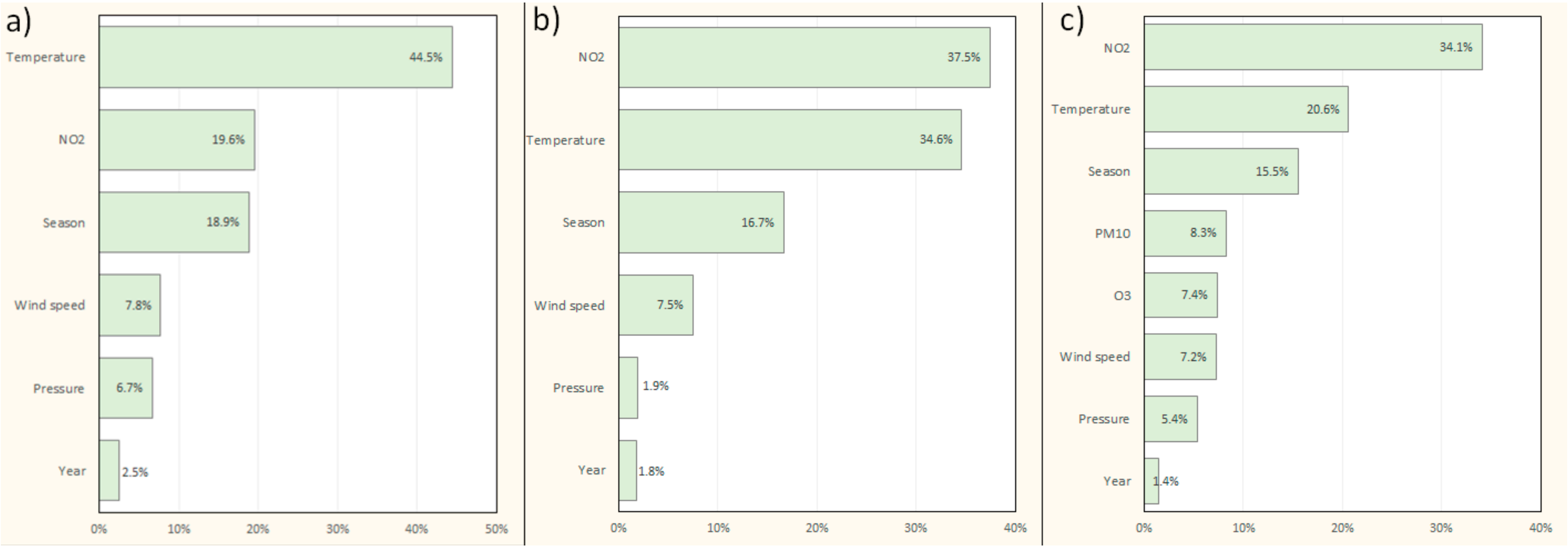

Figure S3. Pareto A graphs showing impact of the most influential individual variables on daily number of pneumonia-related hospitalizations for Warsaw (a), Cracow (b), and Tricity (c). $PM_{2.5}$, particulate matter of size 2.5 µm or less; PM_{10}, particulate matter of size 10 µm or less.

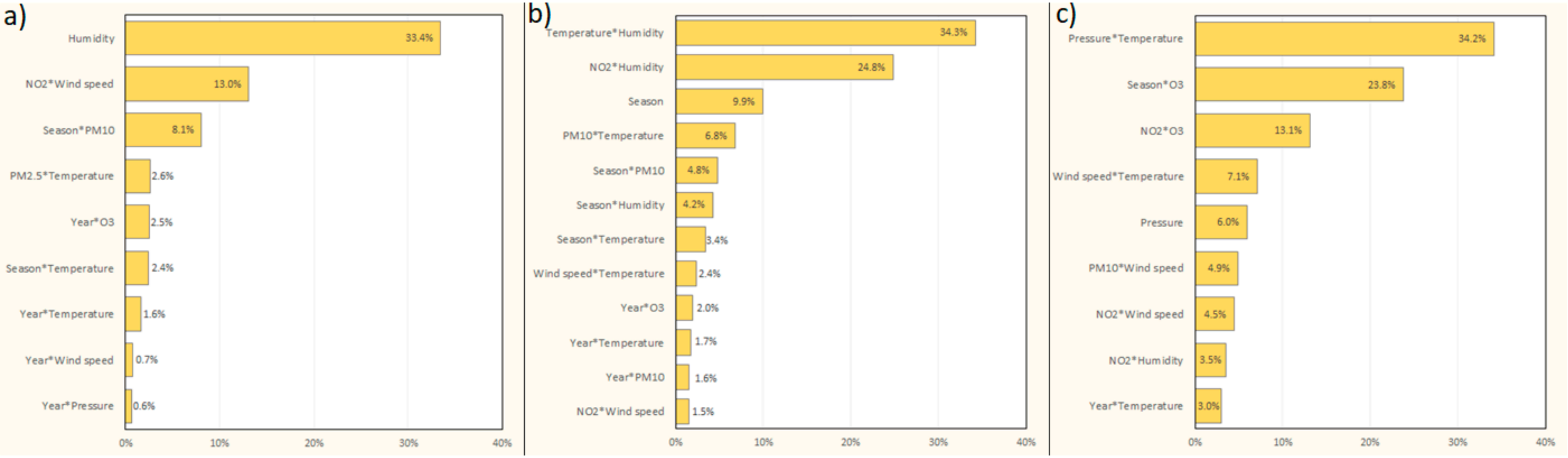

Figure S4. Pareto C graphs showing impact of the most influential (individual or their interactions) variables on daily number of pneumonia-related hospitalizations for Warsaw (a), Cracow (b), and Tricity (c) in final models with interactions. $PM_{2.5}$, particulate matter of size 2.5 µm or less; PM_{10}, particulate matter of size 10 µm or less.
