# Supplementary material for: Influence of Selected Air Pollutants on Mortality and Pneumonia Burden in Three Polish Cities over the Years 2011–2018
Source: J Clin Med. 2022 May 30;11(11):3084. doi: 10.3390/jcm11113084 (PMC9181391; doi:10.3390/jcm11113084)
Supplement: Supplementary file 1 [file jcm-11-03084-s001.zip › Supplementary materials (File S1 - description of GLM and GRM models).pdf]

Brief description of the GLM (generalized linear model) and GRM (generalized regression model) link function models.

Unlike other models, a generalized linear regression model is not a model sensu stricto, but a modeling pathway comprising multiple classes of models and estimation methods:

- Simple regression;
- Multiple regression;
- Factor regression;
- Polynomial regression;
- Response surface regression;
- Response surface regression for mixtures;
- One-way ANOVA;
- Main-effects ANOVA;
- Factorial ANOVA ;
- Analysis of covariance (ANCOVA);
- Equal slopes model.

GRM joins all these models and allows for identification of a cause and effect relationship regardless of the measurement scale of independent variables. To sum up the synthetic description of GRM, it is a statistical model, belonging to a family of generalized linear models, for which it was assumed that the explanatory variables affect the dependent variable by only the so-called systematic component:

$$\eta = X^T \beta_\eta$$

where:

X^T—transposition of vector X

The probability distribution of the explanatory variable is determined by the random component of the model:

$$\eta = X^T \beta_\eta$$

The expected value μ of the random component depends on the systematic component as defined by the link function l:

$$\eta = l(\mu)$$

Depending on the choice of link function, different models are obtained. Unknown parameters β are usually estimated using maximum likelihood, quasi-maximum likelihood, or Bayesian methods. General regression family models enable evaluation of not only the impact of individual factors, but also their interactions. The simplified form of GRM for modeling and predicting pollutant concentrations is as follows:

$$
\begin{aligned}
c_{t+1}{}^{W} = g\big(&p_t{}^{W}c_t{}^{W} + p_{t-1}{}^{W}c_{t-1}{}^{W} + \cdots + p_{t-r}{}^{W}c_{t-r}{}^{W} + p_t^{kW}kW_t{}^{W} + \cdots + p_{t-m}^{kW}kW_{t-m}{}^{W} \\
&+ p_t^{vW}vW_t{}^{W} + \cdots + p_{t-m}^{vW}vW_{t-m}{}^{W} + p_t^{teW}teW_t{}^{W} + \cdots + p_{t-m}^{teW}teW_{t-m}{}^{W} \\
&+ p_t^{ciW}ciW_t{}^{W} + \cdots + p_{t-m}^{ciW}ciW_{t-m}{}^{W} + p_t^{wiW}wiW_t{}^{W} + \cdots + p_{t-m}^{wiW}wiW_{t-m}{}^{W} \\
&+ p_t^{prpW}prpW_t{}^{W} + \cdots + p_{t-m}^{prpW}prpW_{t-m}{}^{W} + p_t^{prmW}prmW_t{}^{W} + \cdots \\
&+ p_{t-m}^{prmW}prmW_{t-m}{}^{W} + p_t^{opW}opW_t{}^{W} + \cdots + p_{t-m}^{opW}opW_{t-m}{}^{W} + p_t{}^{W}WDay(i)_t{}^{W} \\
&+ \cdots + p_{t-m}{}^{W}WDay(i)_{t-m}{}^{W} + p_t{}^{W}HH(ii)_t{}^{W} + \cdots + p_{t-m}{}^{W}HH(ii)_{t-m}{}^{W}\big)^{W} + e_t{}^{W} \\
&+ e_{t-1}{}^{W} + \cdots + e_{t-r}{}^{W}
\end{aligned}
$$

where:

$c_{t+1}{}^{W}$—predicted concentration of the air quality index at station W at time t+1 for t=1 to 505 hours counting backwards from the moment (date and time) of making the model and prediction (t+1, t+2,….,t+48)

$c_t{}^{W}, c_{t-1}{}^{W}, \dots, c_{t-r}{}^{W}$ —measured concentration of the air quality index at station W at time *t, t-1,…, t-r*, where r is the length of the time series determined at the identification stage (r=505);

$p_t{}^{W}, p_{t-1}{}^{W}, \dots, p_{t-r}{}^{W}$ —estimated parameters of concentrations of the air quality index at station W at time *t, t-1,…, t-r,*;

$WDay(i)_t{}^{W}, \dots, WDay(i)_{t-r}{}^{W}$ —variables identifying the weekly cycle after and from 1 to 7 at station W at time t, where r is the length of the time series determined at the identification stage; each day of the week (from 1 to 7) is represented by a 0-1 independent variable;

$HH(ii)_t{}^{W}, \dots, HH(ii)_{t-r}{}^{W}$ —variables identifying the daily cycle of changes (traffic and photochemical transformations) after and from 0:00 to 23:00 at station W at time t, where r is the length of the time series determined at the identification stage;

k—wind direction, *v*—wind speed, *te*—temperature, *ci*—pressure, *prp*—vertical speed, *prm*—sunlight, *op*—precipitation, *wi*—humidity;

$e_t{}^{W}$ —residuals of models at station W;

$g()^{W}$ —model link function selected depending on the distribution of the random component (normal, gamma, Poisson).

Depending on the assumed distribution of the variable y (c_t in the simplified form of GRM) and the measurement scale, different link functions can be used: normal distribution, gamma, inverse normal and Poisson distribution (examples of link functions are presented below):

Identity link function: f(z)=z

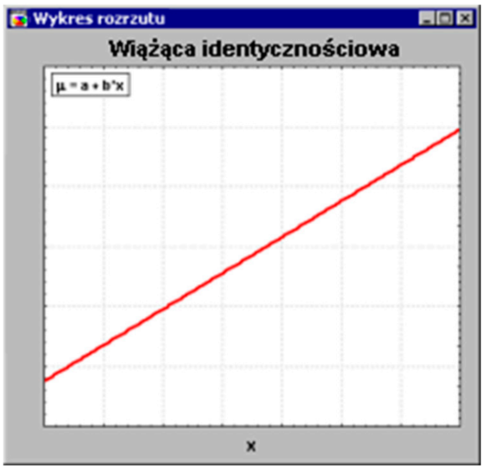

Logarithmic link function: f(z)=log(z)

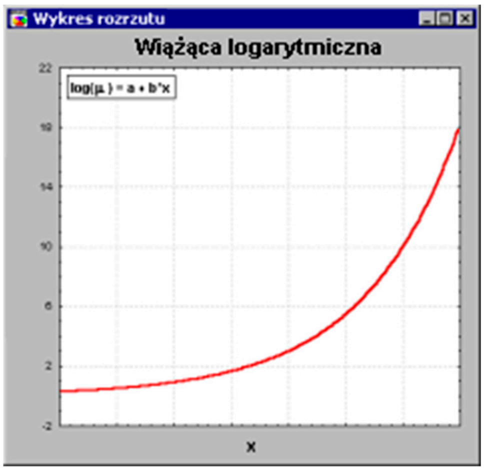

Power link function: f(z)=za, for a given a

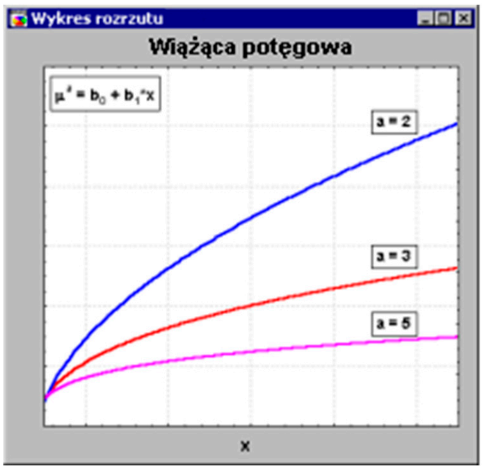

Binomial and multinomial ordinal distributions

Logit link function: f(z)=log(z/(1-z))

Probit link function: f(z)=invnorm(z)

where:

invnorm—inverse of standard normal cumulative distribution.

Complementary log-log link function: f(z)=log(-log(1-z))

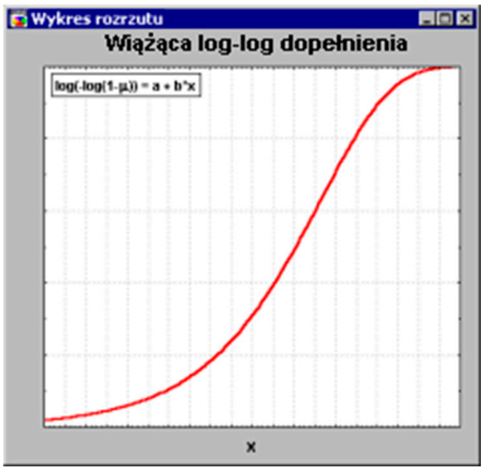

Log-log link function: f(z)=-log(-log(z))

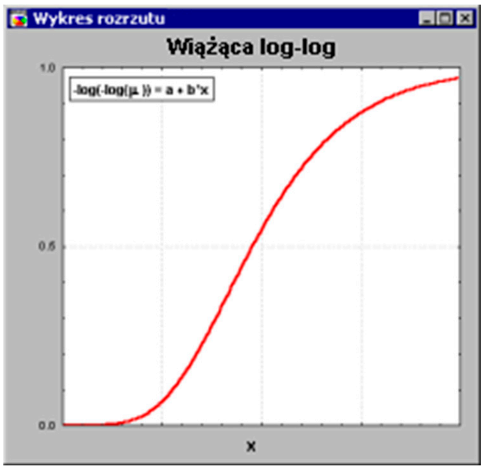

Multinomial distribution
