# Supplementary material for: Influence of Selected Air Pollutants on Mortality and Pneumonia Burden in Three Polish Cities over the Years 2011–2018
Source: J Clin Med. 2022 May 30;11(11):3084. doi: 10.3390/jcm11113084 (PMC9181391; doi:10.3390/jcm11113084)
Supplement: Supplementary file 1 [file jcm-11-03084-s001.zip › Supplementary materials (table S1 - annual mean concentrations).pdf]

**Table S1.** Annual mean concentrations of air pollutants in Warsaw, Krakow and the Tricity, with annual limit values according to the EU regulations and WHO guidelines. Concentrations exceeding EU limit values are in red.

| Air pollutant | Year | Annual mean concentration [µg/m³] | | | EU annual limit value [µg/m³] | WHO guideline [µg/m³] |
|---|---|---|---|---|---|---|
| | | Warsaw | Krakow | Tricity | | |
| $NO_2$ | 2010 | 32.8 | 46.2 | 17.4 | 40.0 | 40.0 |
| | 2011 | 33.1 | 45.3 | 16.1 | | |
| | 2012 | 28.7 | 43.2 | 16.1 | | |
| | 2013 | 38.3 | 40.0 | 14.0 | | |
| | 2014 | 35.4 | 38.1 | 15.7 | | |
| | 2015 | 38.3 | 40.9 | 14.8 | | |
| | 2016 | 37.1 | 41.1 | 15.2 | | |
| | 2017 | 34.4 | 41.1 | 14.1 | | |
| | 2018 | 34.5 | 40.4 | 15.5 | | |
| $O_3$ | 2010 | 41.0 | 32.1 | 48.1 | N/A | N/A |
| | 2011 | 42.6 | 31.3 | 45.8 | | |
| | 2012 | 42.9 | 34.3 | 44.9 | | |
| | 2013 | 43.7 | 34.2 | 51.2 | | |
| | 2014 | 40.2 | 32.7 | 44.5 | | |
| | 2015 | 43.1 | 38.2 | 50.0 | | |
| | 2016 | 40.1 | 33.7 | 47.1 | | |
| | 2017 | 40.8 | 38.2 | 50.0 | | |
| | 2018 | 43.5 | 41.1 | 48.5 | | |
| $PM_{10}$ | 2010 | 33.1 | 61.2 | 22.6 | 40.0 | 20.0 |
| | 2011 | 32.8 | 64.8 | 19.7 | | |
| | 2012 | 33.1 | 59.2 | 18.7 | | |
| | 2013 | 30.5 | 46.5 | 17.2 | | |
| | 2014 | 31.7 | 48.0 | 19.5 | | |
| | 2015 | 32.1 | 48.2 | 16.6 | | |
| | 2016 | 32.9 | 37.9 | 17.2 | | |
| | 2017 | 33.5 | 41.8 | 16.1 | | |
| | 2018 | 38.0 | 38.8 | 21.2 | | |
| $PM_{2.5}$ | 2010 | N/D | 49.7 | 20.5 | 25.0 | 10.0 |
| | 2011 | 29.0 | 44.8 | 19.1 | | |
| | 2012 | 28.5 | 40.8 | 14.0 | | |
| | 2013 | 27.0 | 37.6 | 13.0 | | |
| | 2014 | 26.4 | 36.2 | 16.2 | | |
| | 2015 | 22.9 | 36.4 | 12.9 | | |
| | 2016 | 23.2 | 29.1 | 10.2 | | |
| | 2017 | 25.3 | 33.2 | 9.7 | | |
| | 2018 | 21.6 | 28.5 | 16.4 | | |

EU – European Union, N/A – not applicable, N/D – lack of data
