# Supplementary material for: Influence of Selected Air Pollutants on Mortality and Pneumonia Burden in Three Polish Cities over the Years 2011–2018
Source: J Clin Med. 2022 May 30;11(11):3084. doi: 10.3390/jcm11113084 (PMC9181391; doi:10.3390/jcm11113084)
Supplement: Supplementary file 1 [file jcm-11-03084-s001.zip › Supplementary materials (table S2 - models with lags).pdf]

Table S2. Characteristics of the final models (with interactions and lags) describing effects of air pollutants and weather conditions on the number of all-cause deaths and pneumonia-related hospitalizations. Lags are marked in bold.

Descr.	*Pneumonia*	*Death*	*Pneumonia*	*Death*	*Pneumonia*	*Death*
**ICD10**	**J12_18]**	**TRJ_Death_all**	**WAW_J12_18]**	**WAW_Death_all**	**J12_18]**	**KRA_Death_all**
**city**	TRJ	TRJ	WAW	WAW	KRA	KRA
**A. R^2(inter. Table 2, 3)**	17.4%	12.7%	45.8%	24.0%	29.8%	9.9%
**B. R^2(inter. and lags)**	**19.3%**	**13.1%**	**48.6%**	**25.3%**	**31.9%**	**10.6%**
**B-A**	**1.9%**	**0.4%**	**2.8%**	**1.3%**	**2.1%**	**0.7%**
**Interactions**	SS*TRJ.O3.lag2	SS	WAW.NO2*WAW.TEMP	WAW.O3*WAW.TEMP	KRA.TEMP*KRA.PRES	KRA.PM10*KRA.WV
and lags	TRJ.NO2*TRJ.O3	SS*TRJ.PM10	**WAWNO2.lag2*WAW.PRES**	WAW.TEMP*WAW.PRES	KRA.NO2	SS
	**TRJ.TEMP*TRJ.PM25.lag2**	YYYY*TRJ.PRES	**WAWNO2.lag3*WAWPM25.lag1**	SS*WAW.PM25	**KRA.PM25.lag2*KRA.WV**	**KRA.NO2.lag1*KRA.O3.lag2**
	TRJ.TEMP*TRJ.PRES	TRJ.TEMP*TRJ.PRES	WAW.NO2*WAW.WV	WAW.PM25*WAW.WV	SS	KRA.PRES
	TRJ.PRES	**TRJ.O3.lag2*TRJ.PM10.lag1**	**WAWPM25.lag2**	**WAW.PM25*WAWNO2.lag3**	KRA.TEMP*KRA.WV	KRA.TEMP*KRA.WV
	**TRJ.O3.lag2*TRJ.PM25.lag1**	TRJ.PM25	SS*WAW.PM10	**WAWO3.lag1*WAWPM25.lag1**	**KRA.O3.lag2*KRA.PM25.lag1**	YYYY*KRA.PM10
	**TRJ.O3*TRJ.PM10.lag1**	YYYY*TRJ.TEMP	**WAW.NO2*WAWNO2.lag2**	WAW.NO2*WAW.TEMP	SS*KRA.PM10	YYYY*KRA.WV
	**TRJ.TEMP*TRJ.NO2.lag1**	**TRJ.O3.lag1*TRJ.PM25.lag2**	**WAWNO2.lag1*WAW.WV**	**WAW.O3*WAWNO2.lag2**	**KRA.NO2.lag1*KRA.WV**	**YYYY*KRA.PM10.lag1**
	TRJ.PM25*TRJ.O3		**WAW.NO2*WAWPM25.lag3**	SS	KRA.PM10*KRA.TEMP	
	**TRJ.TEMP*TRJ.NO2.lag2**		SS	SS*WAW.PRES	KRA.WV*KRA.RAIN	
	YYYY*TRJ.TEMP		**SS*WAWNO2.lag2**	**SS*WAWO3.lag3**	YYYY*KRA.TEMP	
	YYYY*SS		SS*WAW.PRES	SS*WAW.O3	**KRA.NO2.lag2*KRA.PRES**	
	YYYY		**YYYY*WAWPM25.lag3**	**WAW.O3*WAWNO2.lag1**	SS*KRA.TEMP	
	YYYY*TRJ.O3		**WAW.NO2*WAWPM10.lag2**	YYYY*WAW.TEMP	KRA.NO2*KRA.WV	
	**YYYY*TRJ.PM10.lag2**		**WAWPM10.lag1*WAWPM10.lag3**	YYYY*WAW.PM10	KRA.O3*KRA.PM10	
			**SS*WAWPM10.lag3**	YYYY*WAW.WV	KRA.PM10*KRA.RAIN	
			YYYY*WAW.O3	**YYYY*WAWNO2.lag2**	YYYY*KRA.O3	
			**WAWO3.lag2*WAW.WV**		YYYY*KRA.WV	
			YYYY*WAW.WV		**YYYY*KRA.NO2.lag2**	
			**WAWNO2.lag3*WAWPM25.lag2**		YYYY*KRA.PM25	
			**WAWNO2.lag3*WAW.PRES**		YYYY*KRA.PM10	
			YYYY*WAW.TEMP		**YYYY*KRA.O3.lag1**	
