# Supplementary material for: Influence of Selected Air Pollutants on Mortality and Pneumonia Burden in Three Polish Cities over the Years 2011–2018
Source: J Clin Med. 2022 May 30;11(11):3084. doi: 10.3390/jcm11113084 (PMC9181391; doi:10.3390/jcm11113084)
Supplement: Supplementary file 1 [file jcm-11-03084-s001.zip › Supplementary materials (table S3 - preliminary lags results).pdf]

Table S3. Preliminary lag identification results from lag 1 to 7 using GRM models.

pollutant	*lag*	*Death*	*Death*	*Death*	*lag*	*Pneumonia*	*Pneumonia*	*Pneumonia*
		**TRJ_Death_all**	**WAW_Death_all**	**KRA_Death_all**		**J12_18]**	**WAW_J12_18]**	**J12_18]**
	**city**	TRJ	WAW	KRA		TRJ	WAW	KRA
**NO_2**	1	0.046	0.034	0.031	1	0.015		0.020
	2				2			
	3	0.028	0.046		3	0.011		
	4				4			
	5				5			
	6	0.040			6	0.013		0.021
	7		0.030		7	0.032	0.073	0.023
**PM_{10}**	1	0.039	0.076	0.010	1		0.039	0.012
	2				2		0.023	
	3		0.033		3	0.008		0.007
	4			0.010	4		0.033	
	5				5			
	6	0.018			6	0.009	0.031	0.014
	7		0.057		7	0.010	0.051	0.006
**$PM_{2.5}$**	1	0.038	0.070	0.011	1	0.009	0.054	0.016
	2				2		0.036	
	3		0.036		3	0.008		0.009
	4			0.010	4		0.032	
	5				5		0.027	
	6				6		0.040	0.017
	7		0.064	0.007	7	0.019	0.059	0.007
**O_3***	1	0.029	0.034	0.036	1			
	2				2	0.008	0.019	
	3				3			
	4				4			
	5				5			
	6				6			
	7				7			

*In case of O3 only the data for the non-heating season have been taken in the analyses

The table shows the parameter values that we interpret as an increase in the concentration of e.g. NO_2 by 1 unit resulting in the number of deaths increases on average by the value of the parameter. The results can be multiplied by scalars x10 or x100. As NO_2 increases by 10 $\mu g/m^3$, the number of deaths increases on average by 0.46 (0.5).
